# Hearing Impairment in French Merchant Seafarers: Retrospective Study on Data from 8308 Audiometric Tests

**DOI:** 10.3390/ijerph19148796

**Published:** 2022-07-20

**Authors:** David Lucas, Thierry Sauvage, Anne Sophie Forestier, Richard Pougnet, Greta Gourier, Brice Loddé, Dominique Jégaden

**Affiliations:** 1 Seamen’s Health Service, Ministry of Transport, F-92040 Paris, France; thierry.sauvage@developpement-durable.gouv.fr; 2 French Society of Maritime Medicine Brest, F-29200 Brest, France; brice.lodde@chu-brest.fr (B.L.); dominique.jegaden@wanadoo.fr (D.J.); 3 ORPHY Laboratory, University Brest, F-29200 Brest, France; 4 Research and Clinical Investigation Unit, Teaching Hospital, F-29200 Brest, France; greta.gourier@chu-brest.fr; 5 Occupational Diseases Center, Teaching Hospital, F-29200 Brest, France; anne-sophie.forestier@chu-brest.fr (A.S.F.); richard.pougnet@chu-brest.fr (R.P.)

**Keywords:** merchant seafarers, hearing impairment, noise-induced hearing loss, occupational epidemiology

## Abstract

Background: A high level of occupational noise exposure has been noted in the fishing sector. Yet, less is known regarding other navigation groups, such as merchant seafarers, since a French study in the 1980s. This study assesses hearing impairment (HI) in a French merchant seafarers’ population. Methods: We collected data of all audiograms performed in 2018 and 2019 for French merchant seafarers. For each seafarer, hearing ability was measured in both ears using pure-tone audiometry at the following frequencies: 0.5, 1, 2, 3, 4, 6, and 8 kHz. Hearing threshold levels (HTLs), or the intensity of sound below which no sound is detected, were measured in decibels Sound Pressure Level (dB SPL) at each frequency and recorded in 5 dB increments. For HI, we used the validated definition of the American Speech–Language–Hearing Association (ASHA). Results: We were able to include statistical analysis results of 8308 audiograms. In a multiple logistic regression adjusted for age, experience, and class of navigation, we found that experience of more than 14 years Odds Ratio OR 1.28 (CI 95% 1.07–1.53), age 31–40 OR 2.2 (CI 95% 1.4–3.4), and >40 years OR 14, 3 (IC 95% 9.7–21) and marine engineers OR 1.26 (IC 95% 1.01–1.57) were still risk factors for HI. Conclusion: In 2018, Marine engineers were still the workers’ group with a higher risk of HI in merchant seafarers but, notch at 4 Hz, specific of noise-induced hearing loss, has improved. They have an HI close to the definition of socioacousis and mean deficit differences with deck and services’ merchant seafarers improved. Our results could be interpreted as a limitation of occupational noise exposure impact in a merchant seafarers’ population, needing an improvement in prevention measures and also encouraged to continue to improve onboard working conditions.

## 1. Introduction

Noise-induced hearing Loss (NIHL) is mainly linked to impulse and continuous noise exposure on the levels that can cause damage to the hearing system [1]. World wide, it is estimated that 1.3 billion people suffer from NIHL, and occupational noise exposure is responsible for 16% of cases of disabling hearing loss in adults. It is an irreversible disease with no effective treatment [2]. Hearing loss at 4 and 6 Kilohertz (kHz) frequencies could limit communication between workers and lead to risks to workers’ safety and performance. It can also lead to increased social stress, sadness, diminished confidence, and poor self-identity [3]; NIHL was defined by a set of criteria applied to audiometric analyses of the National Health and Nutrition Examination Surveys (NHANES) participants from 1999 to 2004 on the basis of an audiometric notch at 4 kHz [4]. An audiometric notch maximal at 3, 4, or 6 kHz with an improvement at 8 kHz is typical of NIHL but can be obscured by age-related hearing loss, also called presbycusis [5].

For a seafarers’ population, some risk factors of HI have been highlighted in the literature, including working in engine rooms, described in a Danish cohort published in 2008, which showed that fishermen and seafarers working in engine rooms were more likely to consult a hearing clinic than other seafarers and the general population [6]. Indeed, age and time in the profession have been noted as such factors. Firstly, in a study on occupational noise exposure, including 18,109 seafarers, the percentage of seafarers with a hearing loss of at least 30 dB SPL increased with age according to an exponential curve, reaching 28% for the 50–55 age group [7]. Moreover, fishermen and seafarers can be exposed to many different sources of high-level noises within their work shifts, usually longer than 8 h per day. These studies showed that seafarers working in engine rooms have a higher risk of HI and also fishermen are exposed to developing NIHL and, of course, like all workers, to presbycusis.

A French study carried out by Jégaden in the 1980s on 222 seafarers found a level of noise exposure above the regulatory standards with a variable impact on the seafarers’ hearing.

This study showed a typical hearing impairment with a notch at 4 kHz for marine engineers, while the deck crew had early presbycusis-like impairment which can be likened to socioacusis [8]. Socioacusis is defined as the hearing loss produced by exposure to nonoccupational noise in combination with lifestyle factors (such as hearing loud music, using power tools at home without hearing protection, etc.) [1,9].

Indeed, a study carried out in 2016 on 227 professional fishermen showed that a high rate of fishermen worked for long periods (more than 16 h), with noise levels ranging from 94.8 to 105.0 dBA. A distinction was made between the number of people with hearing impairment (which we could relate to presbycusis) and those with NIHL. The results showed, on one hand, that the number of people with NIHL was significantly correlated with the length of time in the fishing profession but not with age. On the other hand, the number of hearing-impaired people was significantly related to age but not to the number of years spent in the trade [10].

Recommendations to limit occupational exposure levels have been published at the international and French levels [11,12]. New safety legislation has resulted in lower noise exposure in engine rooms, better technical innovations, and enclosure systems have improved occupational exposure levels. Training courses have provided better knowledge and awareness of the risk. The use of personal protective equipment has strongly increased the safety in the occupational maritime field.

Hearing impairment studies in seafarers and fishermen are scarce and have only been conducted in a few geographical areas [13,14,15,16,17,18].

The objectives are to investigate the hearing impairment prevalence in merchant French seafarers, to assess the impact of occupational noise exposure and the risk factors, and to follow the evolution in comparison to Jégaden‘study carried out in the 1980s up till now [8].

## 2. Materials and Methods

We performed a cross-sectional observational study using retrospective data from the Health at Work file.

In France, a specific occupational prevention unit called the Service de Santé des Gens de Mer (SSGM) oversees the medical examination of French seafarers. As part of the monitoring of occupational noise exposure and to assess the impact on the hearing capacities of seafarers, they carried out annual or biannual audiograms. The Labour Code stipulates that a worker whose daily exposure to noise exceeds 80 dBA or 135 dBC peak sound pressure level may, at his or her request or that of the occupational physician, undergo a screening audiometric test [11,19,20]. Audiometric tests were performed by trained occupational nurses or occupational physicians with the same protocol in each SSGM. Most of the SSGMs are provided with sound-treated booths. For each seafarer, hearing ability was measured in both ears using pure-tone audiometry at the following frequencies: 0.5, 1, 2, 3, 4, 6, and 8 kHz. Hearing threshold levels (HTLs), or the intensity of sound below which no sound is detected, were measured in decibels Sound Pressure Level (dB SPL) at each frequency and recorded in 5 dB increments.

The data from audiometry are directly put into the seafarer’s occupational health file during medical examination at the SSGM. The data from the audiometric tests were anonymously extracted by the SSGM’s Informatics’ department. As audiometry is carried out at one or two-year intervals, we included all audiograms from 2018 and 2019. To avoid duplication, when two audiograms existed for the same file reference, most recent was included. Data from all the occupational health centers of the SSGM in France were included.

### 2.1. Inclusion Criteria

Inclusion criteria: French registered merchant seafarer and audiometric test in 2018 or 2019 at the SSGM. As for the non-inclusion criteria: patients under judicial protection.

### 2.2. Data Privacy

During the examination at the SSGM, clear information on data extraction and used for research was given to seamen and they could decline it. These data are collected as part of mandatory health assessments. It is written in information form that non-identified data could be used for research. All data were de-identified before extraction and analysis. We ensured that the data were kept anonymous and secure.

Hearing impairment (HI) was defined using American Speech–Language–Hearing Association (ASHA), as an average HTL among frequencies 1, 2, 3, and 4 kHz more than 25 dB SPL in either ear [21].

### 2.3. Statistical Analysis

The characteristics of the patients were described by number and frequency with percentage for the qualitative variables.

Duration was divided into 2 classes (≤13 years, >13 years), age into 3 classes (<30, 30–39, >40 years), merchant seafarers’ population in 3 groups: marine engineer, deck, and service.

Merchant seafarer’s group: international cabotage towing, international cabotage, pilotage, national cabotage towing, coastal navigation towing, national cabotage, coastal navigation, coastal navigation towing, long-distance towing, and long distance.

For HI, we used a cut-off at 25 dB, with a class normal (≤25 dB) and abnormal [19].

Descriptive statistics were calculated from the survey results and presented as counts and percentages. In total, 34,208 audiometric tests were extracted. After excluding due to incomplete or missing survey or audiogram data (6 or 8 kHz in majority) from other navigation groups, 8308 audiometric tests were included in the data analyses.

For each audiometric frequency tested, mean HTLs were presented by age group and workplace categories. Chi-squared tests were used to examine the relation between NIHI and categorial variables such as workplace categories, experience, and age.

Measures of association were computed from 2 × 2 contingency tables and reported as odds ratio (OR) with a 95% confidence interval (CI). Seafarers’ workplace categories (engine room, deck, and services) as independent risk factor for HI were examined, and these results were adjusted for age and years of experience to control for its confounding effect. For reference group, we used, respectively, HI ≤ 25 dB fo HI, age < 30 years for age, experience ≤ 13 years for experience, and service for workplaces’ categories adjusted OR and 95% CI were calculated according to the Cochran–Mantel–Haenszel method. A *p* value less than 0.05 was considered statistically significant. Data analyses were conducted using XL stat software 2020 (version 2, Addinsoft Corporation, New York, NY, USA).

## 3. Results

We collected data from 34,208 audiograms of all French seafarers. Tests with missing thresholds at 0.5, 1, 2, and/or 4 kHz were removed from the analysis, and we were able to include 8308 audiograms for merchant mariners in the statistical analysis.

### 3.1. Main Characteristics

The main characteristics of the total population are summarized in Table 1. Most of them are male (82.7%), older than 41 years (46.4%), have less than 13 years of experience (58%), and have worked on the deck (43.4%). Mean age in years of groups of navigation are, respectively, for machine engineers, desk, and service seafarers 39 ± 10.7; 41.2 ± 10.8, and 38.9 ± 11.5. For experience at work, it was in years 17.1 ± 11.5; 17.2 ± 12.6 and 9.3 ± 10.6. Mean of age and experience are summarised in Table 2.

Our results showed that most of our population is under 40 years of age and has been in the workforce for less than 13 years. As we know that the auditory impact of noise is most often revealed beyond the age of 40, and often even later, we will study, in more detail, the hearing losses of the latter age and seniority categories.

### 3.2. Average Hearing Threshold

**Figure 1:**
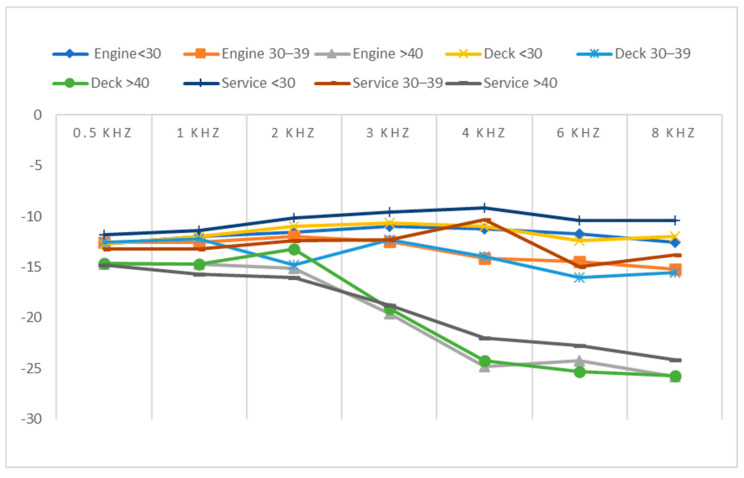
Graphic of mean hearing threshold levels in the sample of merchant seafarers, in each frequency tested.

The average hearing threshold levels at all tested frequencies, categorized by age and location, are summarized in Table 3 and Figure 1.

**Figure 2:**
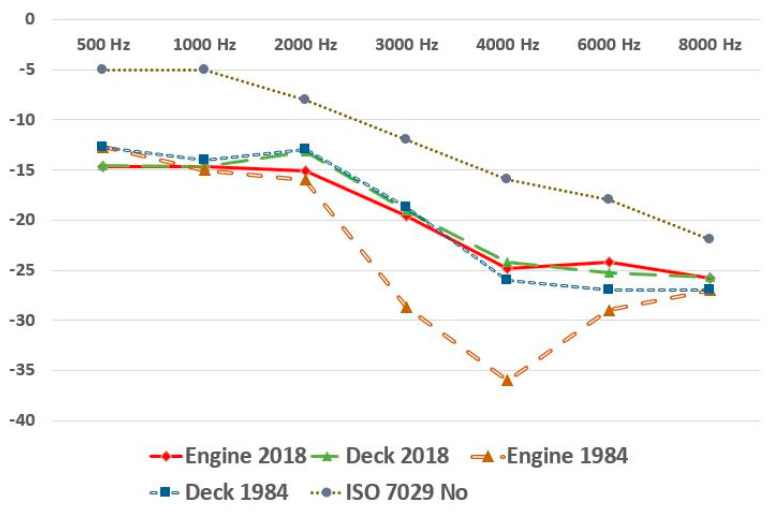
Comparison of median audiograms of seafarers over 40 year timespan (deck/marine engineers) between 1984 data (Jegaden), 2018–2019 data from our study, and the normal physiological audiometric curve according to ISO 7029 corresponding to the age class.

Comparison of average HTLs of seafarers over a 40 year timespan (deck/marine engineers) between 1984 data (Jegaden), 2018–2019 data from our study, and the normal physiological audiometric curve according to ISO 7029 corresponding to the age class is shown in Figure 2.

We note that the values of the deficits, at each frequency, are very close in each age group and in relation to workplace—deck, engine room, or service—the latter being slightly less impacted than the other two. 

### 3.3. Univariate and Multivariate Analysis

For the HI analysis, univariate and multivariate analyses are reported in Table 4. For non-normal HIs, in univariate analysis, years of experience greater than 14 years OR 3.28 (CI 95% 2.81–3.83), age, respectively, for 31–40 and <41 years OR 2, 5 (CI 95% 1.7–3.8) and 17.1 (CI 95% 11.9–24.6) are risk factors and with reference to merchant services’ seafarers, merchant marine engineers and desk seafarers have a higher risk with, respectively, OR 1.54 (1.25–1.88) and 1.532 (CI 95% 1.28–1.8). In multivariate analysis with binomial regression, risk factors were still experience of more than 14 years OR 1.28 (CI 95% 1.07–1.53), age for the group of 31–40 an OR 2.2 (CI 95% 1.4–3.4); the group > 40 years OR 14, 3 (IC 95% 9.7–21) and also with reference to desk seafarers merchant marine engineers s OR 1.26 (IC 95% 1.01–1.57) were still risk factors for HI in class > 26 dB.

## 4. Discussion

By analysing a large dataset from the routine medical examination of French merchant seafarers with audiometric measurements, we found that age, years of experience, and working in an engine room are risk factors for HI. These results show a very clear improvement, over time, in the hearing of the merchant marine engineers of merchant ships. The results confirm previous studies, showing a progressive hearing impairment with age and seniority, consistent with the fact that ship engine rooms are a very noisy place, which is attested by numerous sonometric studies [22].

### 4.1. Interest of the Study

In one way, an interesting point of our study is to confirm, with a large number of audiograms, risk factors of HI in merchant seafarers. In another way, an interesting point of our study is to compare 1985 with ours. Our study is the only complete audiometric study on hearing loss in merchant seafarers comparable to the Jegaden study about 40 years ago [8]. The Jégaden study showed a typical noise-induced hearing loss audiometric curve in merchant marine engineers over 40 years of age, with a clear notch at the frequency 4 kHz of the order of more than 30 dB and a rise at frequencies 6–80 kHz. This notch at 4 kHz was not found in deck seafarers over 40 years of age, sailing on the same type of ships. They showed a moderate and progressive hearing loss between 3 and 8 kHz, suggesting socioacusis [23]. In a 1979 study on merchant ships, Schmidt and Gales et al. noted that the 24 h average equivalent levels (Leq24) of marine engineers were significantly higher than those of non-engine-room crew members. Marine engineers’ equivalent levels ranged from 78 dBA to 96 dBA, while non-engine-room crew members ranged from 57 dBA to 71 dBA [24]. These data could explain the audiometric results of Jégaden’ study.

However, in our study, the audiogram-like presentation of the mean HTLs of French seafarers included between 2018 and 2019 no longer shows this typical notch at 4 kHz, but a curve similar for deck seafarers. It is, of course, difficult to compare studies that are very far apart in time based on different numbers of workers and done with different methodologies. Nevertheless, we have this approach between the audiometric graphs of Jégaden and ours, more specifically, by superimposing the curves of young seafarers aged 16 to 30 years and by comparing the age-related curve modifications for the other seafarers’ populations.

We then see very clearly (Figure 2) that, on the one hand, the curve for the deck seafarers of 1984 (blue line) is superimposable on that of the deck seafarers of 2018 (green line), which introduces the idea of a constancy of socioacusis over time in this category of seafarers subjected to noise levels in the order of 70 dBA already in the 1980s, which corresponds very well to the mentioned definition of socioacusis. These curves have a similar slope to the normal physiological curve given by the ISO 7029-2017 standard for this age group, but with a greater level of deficit than the latter [25]. On the other hand, and this is the new and interesting character of our work, we note, in the current (2018–2019) merchant marine engineers (red line), a fading of the notch at 4 kHz, which was very clear in 1984 (brown line). Indeed, the curve for the marine engineers is close to deck seafarers in 1984 (brown line) and 2018 (green line). These results show clear improvements, over time, in the hearing of merchant marine engineers of merchant ships, which is good news for seafarers’ health. They illustrate the impact of the international regulations on noise levels onboard ships (IMO Resolution A 468 XII of 1981, European Directive 2003/10/EC and more recently MSC Resolution 337 (911) of 2012 with effect from 1 July 2014) [26,27,28]. The new regulations limit noise levels to 75 dBA maximum in machinery control rooms. Analysis of recent ship noise data by Bocanegra et al. yields an average noise level of 69 dBA (±13) on board current ships, with only 10% of measurements exceeding the 85 dB level [29]. Although engine rooms remain very noisy (110 dBA), the automation of ships has significantly reduced the time spent in these spaces and greatly increased the time spent in the monitoring control room [30]. As Occupational NIHL is an irreversible disease with no effective treatment, prevention remains the best option for limiting impact of noise exposure. Monitoring noise exposures, reducing noise exposure in workplaces, and early detection of HI are principal purposes for prevention. Now and for the future, ships with electric engines are developed and more and more efficient. Another recent technological innovation with impact on noise exposure comes from pods. Pods are devices that combine both propulsive and steering functions in one device. Several advantages have been attributed to pod propulsion systems, such as: reduced emissions, and lower noise and vibration levels and emissions. In new ships, pods’ engines are located at the stern, far from the occupational environment of seafarers. In addition, all merchant marine engineers now routinely wear ear protection for several decades while working in the engine room. It is also possible that the reduction in embarkation time, currently 2 months for 2 months of leave on average, has a long-term beneficial influence on hearing loss in these seafarers. Providing appropriate hearing protective devices and instructing workers to use them are also important strategies for prevention.

A major strength of our study is the inclusion of a large number of seafarers from a national database. The large number of data included allowed us to perform a multivariate analysis and increase the validity of our results.

Comparison between two different time periods over a 40 year timespan, including the same type of merchant seafarers, highlighted the importance of HI evaluation in occupational groups with noise exposure. It also underlined the relevance of testing the impact on occupational health in the prevention program.

### 4.2. Limitations of the Study

Firstly, in order to eliminate any hearing fatigue, the audiogram must be performed without any occupational exposure to noise for at least 48 h, which is the case for the large majority of commercial seafarers. On the other hand, as not all SSGMs have soundproof booths to perform these measurements, we can question audiograms’ validity. We also do not know methods of result transcription into the computerized medical record. Nevertheless, the consistency of the overall results allows us to consider their good value.

Second, there may be a confounding bias between the effects of noise on hearing and those of other factors, such as smoking, which is frequent among seafarers. Some studies have suggested that smoking directly affects the auditory system through nicotine and other ototoxic substances in cigarette smoke [31]. A meta-analysis published in 2005 concluded that there is a moderate to significant association between smoking and hearing loss [32]. Other co-founders, such as hyperlipidemia [33] and occupational exposure to ototoxic solvents [34], can also be identified in the seafaring population. Combined with noise, they could also, to a lesser extent, impact the hearing of seafarers.

Third, due to the large and representative population of French seafarers, our study results could be generalised to the global population of merchant seafarers. However, some countries still maintain very old and noisy ships and lack the use of hearing protection, having a noise health impact among seafarers, as suggested by Vukić et al.; we are not able to validate this hypothesis [35].

Authors should discuss the results and how they can be interpreted from the perspective of previous studies and of the working hypotheses. The findings and their implications should be discussed in the broadest context possible. Future research directions may also be highlighted.

## 5. Conclusions

If age, seniority in the profession, and the fact of working in the merchant ships engine room remain risk factors to noise-induced hearing loss, it is interesting to underline that this noise-induced hearing loss, typical in 40-year-old studies among marine engineers, has considerably improved. Stricter regulations in ship soundproofing and the change in the types of propulsion, in particular with less noisy new diesel-electric systems, could explain it. Engineers now have a hearing impairment, of the socio-acoustic type, very close to deck and service seafarers. Nevertheless, these results remain to be confirmed by other studies, due to limitations expressed about the uncontrolled and rigorous collection of data.

## Figures and Tables

**Table 1:** Demographic characteristics of included population.

	Total of Serafers		Number	Frequency (%)
Sexe	8308	Men	6874	82.7
		Women	1434	17.3
age in years	8308	<30	2054	24.7
		30–39	2396	28.8
		>40	3858	46.4
Experience in years	8308	≤13	4821	58
		>14	3487	41.9
Service	8308	Engine	1892	22.8
		Deck	3612	43.4
		Service	2804	33.7

**Table 2:** Mean of age and experience in years of included population.

	Navigation Group	Mean	Standard Deviation	*t* Test *p* Value
**AGE**	Engine	39	10.7	
	Desk	41.2	10.8	<0.001
	Service	38.9	11.5	0.66
**EXPERIENCE**	Engine	17.1	11.5	
	Desk	17.2	12.6	0.64
	Service	9.3	10.5	<0.001

The *p* value was derived from *t* test.

**Table 3:** Mean hearing threshold levels in the sample of merchant seafarers, in each frequency tested.

Workplace	Age	0.5 kHz	1 kHz	2 kHz	3 kHz	4 kHz	6 kHz	8 kHz
Engine room	<30	−12.7	−12	−11.6	−11	−11.2	−11.7	−12.6
	30–39	−12.6	−12.6	−12	−12.5	−14.1	−14.5	−15. 2
	>40	−14.7	−14.7	−15.1	−19.6	−24.8	−24.2	−25.8
Deck	<30	−12.7	−12	−11	−10.7	−11	−12.4	−12
	30–39	−12.6	−12.2	−14.8	−12.3	−14	−16	−15.5
	>40	−14.6	−14.7	−13.2	−19.1	−24.2	−25.3	−25.7
Service	<30	−11.8	−11.4	−10.2	−9.6	−9.2	−10.4	−10.4
	30–39	−13.2	−13.2	−12.4	−12.3	−13.3	−15	−13.8
	>40	−14.8	−15.7	−16	−18.8	−22	−22.7	−24.1

**Table 4:** Univariate and multivariate analysis adjusted for co-founders including, age, experience, and workplaces of navigation.

		Hearing Impairment		*p*	OR (IC 95%)	*p*	Adjusted OR (IC 95%)
		>26	≤25				
Age	<30	65	1990		Ref		Ref
	30–39	31	2366	<0.001	2.5 (1.7–3.8)	<0.001	2.2 (1.4–3.4)
	>40	707	3149	<0.001	17.1 (11.9–24.6)	<0.001	14.3 (9.7–21)
Years of experience	≤13	258	4566		Ref		Ref
	>14	545	2939	<0.001	3.28 (2.8–3.8)	<0.001	1.28 (1.07–1.53)
	Engine	206	1687	<0.001	1.54 (1.25–1.9)	<0.01	1.26 (1–1.57)
Workplace	Deck	391	3220	<0.001	1.5 (1.28–1.8)	0.1	1.07 (0.9–1.3)
	Service	201	2598		Ref		Ref

OR: Odds Ratio. IC 95%: Interval of Confidence at 95%. *p*: *p* value.

## Data Availability

Data are located in Brest Teaching Hospital, Unit of research and clinical investigation. Specific requests can be sent to the corresponding author.
